# EMAP II Expression Is Increased on Peripheral Blood Cells from Non-Hodgkin Lymphoma

**DOI:** 10.1155/2022/7219207

**Published:** 2022-09-12

**Authors:** Manal Mohamed Saber

**Affiliations:** Department of Clinical Pathology, Faculty of Medicine, Minia University, Minia, Egypt

## Abstract

Tumor immune evasion is a lineament of cancer. Endothelial monocyte activating polypeptide-II (EMAP II) has been assumed to impact tumor immune escape significantly. EMAP II was first reported in the murine methylcholanthrene A-induced fibrosarcoma supernatant and identified as a tumor-derived cytokine. This study evaluated EMAP II expression in peripheral blood cells and its association with treatment outcome, lactate dehydrogenase (LDH) levels, and clinical criteria in non-Hodgkin's lymphoma (NHL) patients. EMAP II expression on different blood cells obtained from the peripheral blood of 80 NHL patients was evaluated by two-color flow cytometry. The study reported that EMAP II expression was significantly increased in peripheral blood cells in patients with NHL compared to normal volunteers (*P* < 0.001). Additionally, EMAP II expression levels on blood cells decreased in complete remission (CR) while they increased in relapse. This study showed coexpression of EMAP II and CD36 on peripheral lymphocytes in NHL patients but not in healthy controls (*P* < 0.001). EMAP II expression on blood cells was associated with increased serum LDH levels. Furthermore, the percentages of EMAP II+/CD36+ peripheral lymphocytes were significantly higher in relapse than in CR and healthy controls. Analyses revealed that higher percentages of EMAP II+CD36+ cells were positively correlated with hepatomegaly, splenomegaly, and an advanced (intermediate and high risk) NHL stage. The results assume that EMAP II might be involved in NHL development and pathogenesis.

## 1. Introduction

Non-Hodgkin's lymphomas (NHL) are malignant lymphoproliferative diseases characterized by heterogeneous clinical and histological criteria [1, 2]. NHL is manifested by the abnormal accumulation or proliferation of B, T, and natural killer (NK) cells that infiltrate hematopoietic and lymphoid tissues and extend to different organs [3, 4]. Follicular lymphoma (about 10%) and diffuse large B-cell lymphoma (approximately 30%) are the most frequent NHL subtypes, while the frequency of all other NHL subtypes is less than 10% [5]. For the treatment of NHL patients, a slew of new therapeutic protocols based on a cocktail of multidrug chemotherapy has been developed [6]. In aggressive NHL, multidrug treatment results in a five-year overall survival rate of 50–60% [7]. However, many patients relapsed, either due to failure after lengthy treatment, referred to as a refractory disease, or due to relapse after an initial response, referred to as relapsing disease [8]. Thus, the search for reliable markers in NHL might usher in a new era of cancer immunotherapy.

Endothelial monocyte activating polypeptide-II (EMAP II), which is produced from its precursor aminoacyl-tRNA synthetase-interacting multifunctional protein 1 (AIMP1), is expressed as a 34 kDa intracellular peptide [9]. EMAP II mRNA and the corresponding precursor protein, proEMAP, have been found in normal and malignant tissue [10–15]. Full-length cDNAs encoding human and murine EMAP II were isolated from normal peripheral blood cells [12]. AIMP1 enhanced normal macrophages and dendritic cells to release IL-12 and initiate Th1 responses [16, 17]. On the cell surface, the C terminus of pro-EMAP II undergoes proteolytic cleavage by apoptosis and protease inhibitors [18–20] to generate the extracellular 22 kDa C-terminal peptide [21–23] that acts as an antiangiogenic protein [9, 24].

EMAP II is classified as a part of the aminoacyl-tRNA synthetase (ARS) family due to a high degree of similarity between it and ARS p43 [20]. EMAP II and p43 have a high degree of similarity in amino acid sequence, and the human homologs of p43 and EMAP II are congruent. p43's extracellular function is identical to that of EMAP II in terms of its angiogenic properties [25].

EMAP II was first reported in the murine methylcholanthrene A-induced fibrosarcoma supernatant and identified as a tumor-derived cytokine based on its propensity to enhance procoagulant activity in the cultured endothelial cells [21]. It has various activities against neutrophils, macrophages, and endothelial cells [26, 27]. As a result, EMAP II has antiangiogenic and proinflammatory properties [28, 29]. EMAP II is a 169-amino-acid cytokine that plays a role in inflammation, apoptosis, and angiogenesis [30, 31]. Endothelial apoptosis, hypoxia, and cellular stress were observed to induce the processing and release of EMAP II [14, 32–34].

EMAP II not only promotes cancer formation by increasing tumor autophagy, sensitizing tumor cells to tumor necrosis factor- (TNF-) alpha, blocking angiogenesis, and increasing brain tumor barrier permeability [35–44], but also promotes cancer formation by causing lymphocyte death [31, 45–47]. EMAP II expression induced lymphocyte apoptosis, suggesting an immunosuppressive role in cancer [48]. Previous data indicated that serum EMAP II might be a potential biomarker in patients with NHL and lung cancer [49, 50]. Some reports have examined EMAP II expression in tumors and its correlation with prognosis [33, 50, 51].

CD36 (cluster of differentiation 36), a scavenger receptor, is a protein encoded by the CD36 gene and expressed in different human immune cells [52]. CD36 promotes the association of lipid rafts with receptors, adapter molecules, and signaling [53]. It is expressed in tumor cells, where it binds, initiates internalization, and regulates long-chain FAS transport [54, 55]. CD36 has a significant role in cancer progression, spread, and metastasis [56–58]. CD36 expression has been explored in different types of NHL [59, 60].

Different immune checkpoint molecules regulate immunity against cancer. EMAP II is one of the molecules expressed on tumor cells and provides a negative signal by inducing lymphocyte apoptosis in cancer. Some reports have examined EMAP II expression in tumors and its correlation with prognosis. However, EMAP II expression on peripheral blood cells of cancer patients, especially in NHL, has not been studied. This paper analyzed EMAP II expression on peripheral blood cells in NHL patients and revealed some association with prognosis in NHL.

## 2. Subjects and Methods

### 2.1. Subjects

The study was done at the Oncology Department, Minia Oncology Centre, Minia, Egypt, and at the Clinical Pathology Department, Minia University, Faculty of Medicine, Egypt, Minia. Thirty healthy controls were involved in this study and 80 NHL patients. Blood was withdrawn from healthy volunteers without autoimmunity, immunosuppression, or malignancy. NHL subjects were divided into three groups: group I: newly diagnosed NHL subjects and not started therapy, group II: NHL subjects who achieved complete remission (CR), and group III: NHL subjects with relapse. NHL diagnosis was confirmed by histopathology and immunophenotyping. The responses of NHL patients have been evaluated regarding the response criteria of Cheson et al. [61]. CR is recognised by free laboratory data and the absence of radiographic symptoms and signs of the disease. Relapse was identified as disease return within five months of complete remission, lymphoma progression during the first treatment, and failure to have complete or partial remission after therapy [61]. NHL patients and normal controls gave informed written consent.

### 2.2. Clinical Characteristics and Samples Collection

Clinical examination, lymph node biopsy, and bone marrow aspiration defined NHL's stage, type, and clinical evaluation. Three experienced pathologists performed the validation of the pathological specimens according to WHO classifications [62]. A flow cytometer was used to perform immunophenotyping for NHL patients. The individuals were also subjected to an X-ray and a pelviabdominal ultrasound. The status of NHL was evaluated on the Eastern Cooperative Oncology Group scale [63]. The Ann Arbor system was used to determine the NHL stage [64]. NHL subjects with incomplete pathological or clinical information did not participate in this study. The controls with chronic infections or autoimmune diseases were excluded from the study. For flow cytometric analysis, 2 mL of blood was placed in a sterile K3EDTA tube. In addition, 3 mL of blood was centrifuged in a plain tube, and the produced serum was used to measure lactate dehydrogenase (LDH) levels using an automated clinical chemistry analyzer (Schiapparelli Biosystems, Inc.).

### 2.3. NHL Treatment

Twenty newly diagnosed NHL patients have not received treatment yet. 60 NHL patients received CHOP (cyclophosphamide, hydroxydaunorubicin, oncovin, prednisone) therapy [65].

### 2.4. Antibodies

Flow cytometry analysis with a verified EMAP II antibody was used to examine EMAP II expression in peripheral blood cells: 546-2, monoclonal antibody (Santa Cruz; catalog no. 32723); CD3 antibody, UCHT1, monoclonal antibody (BioLegend; catalog no. 300406); CD4 antibody, RPA-T4, monoclonal antibody (BioLegend; catalog no. 300506); CD4 Antibody; RPA-T4, monoclonal antibody (BioLegend; catalog no. 300530); CD8 antibody, SK1, monoclonal antibody (BioLegend; catalog no. 344704); CD16 antibody, 3G8, monoclonal antibody (BioLegend; catalog no. 302006); CD20 antibody, 2H7, monoclonal antibody (BioLegend; catalog no. 302304); CD22 antibody, S-HCL-1, monoclonal antibody (BioLegend; catalog no. 363508); HLA-DR antibody, L243, monoclonal antibody (BioLegend; catalog no. 980402); and CD36 antibody, 5-271, monoclonal antibody (BioLegend; catalog No. 336204). For the negative controls is PE mouse IgG2b (k) antibody, 27-35, isotype control antibody (BioLegend; catalog no. 402203). Blocking, immunoprecipitation, and immunohistochemistry (IHC) were used to confirm each antibody for flow cytometric analysis.

### 2.5. Flow Cytometry Analysis

Flow cytometry analysis was assessed by having the following antibodies: CD4-FITC, CD4-PerCP-Cy5.5, CD3-FITC, CD8-FITC, CD16-FITC, CD20-FITC, CD22-FITC, CD5-FITC, CD7-FITC, CD36-FITC, HLA-DR-FITC, EMAP II-PE, and PE isotype control. Antibodies' staining was assessed according to the instructions of the manufacturer. 5 *μ*L of fluorescently conjugated antibodies were mixed with 100 *μ*L of blood and incubated at room temperature for fifteen minutes in the dark. Subsequently, 2 mL red cell lysis buffer was added (BD FACS lysing solution), vortexed, and then was made to sit for fifteen minutes at room temperature in the dark. Sample centrifugation was performed for about 5 min at 1200 rpm. The supernatant was taken out, and 1 mL of phosphate-buffered saline (PBS) solution was put into each tube and mixed thoroughly. The tubes were centrifuged for five minutes at 1200 rpm, and the supernatant was taken out. To resuspend the cells for flow cytometry analysis, 300 *μ*L PBS was added.

A minimum of ten thousand total events were collected and analyzed [66–68]. Two-color immunofluorescence analysis was performed on BD FACSCanto II (Becton Dickinson, San Diego, CA, USA). The analysis of the data was performed by FACSDiva. To exclude cell aggregates and debris, lymphocytes were gated using the scatter forward (size) vs. side scatter (granularity) technique (FSC/SSC) [69–74]. The percentage of cells stained with antibodies was used to represent the results. The percentages of positive cells were assessed from the lymphocyte gate. According to the isotypic controls, the percentage of positive cells was determined. An isotype-matched control was used to assess background fluorescence. For each subject, unstained cells were used as a negative control.

### 2.6. Statistical Analyses

All statistical analyses were conducted using IBM SPSS Statistics, version 24 (IBM; Armonk, New York, USA). The Shapiro-Wilk test checks normality. Continuous variables were expressed as the mean and standard deviation (SD) if normally distributed or the median and interquartile range (IQR) if not normally distributed. Numbers and percentages were used to present categorical variables. Student's *t*-test or Mann–Whitney *U* test was used to compare two independent groups' variables, as applicable. Kruskal-Wallis was used to compare independent groups for nonparametric data, followed by Dunn's test with Bonferroni correction to assess intergroup differences. The strength of the linear link between two continuous variables was estimated using Spearman's correlation. A *P* value of less than 0.05 was considered significant, and values less than 0.001 were regarded as highly significant.

## 3. Results

### 3.1. Patients' Criteria

NHL patients' criteria are illustrated in Table 1. For 20 NHL patients, peripheral blood samples were taken at early NHL diagnosis. Thirty NHL patients had complete remission of the disease. Thirty patients experienced a relapse. The mean age of NHL subjects was 45.3 ± 15.7 years, while it was 44.5 ± 15 for healthy controls. 80 NHL patients (38 female, 42 male) and 30 normal controls (12 female, 18 male) were involved. Regarding gender and age, healthy individuals and NHL subjects did not reveal any difference (*P* > 0.05) (Supplementary Table 1).

### 3.2. EMAP II Expression on Peripheral Blood Cells from NHL Subjects

High differences were identified between all NHL subjects and normal controls regarding the percentage of EMAP II+CD4+, EMAP II+CD16+, EMAP II+CD20+, and EMAP II+CD22+ (*P* < 0.001). However, no differences were found between NHL subjects and normal volunteers regarding EMAP II+CD8+ percentages (*P* = 0.911) (Supplementary Table 2).

**Figure 1:**
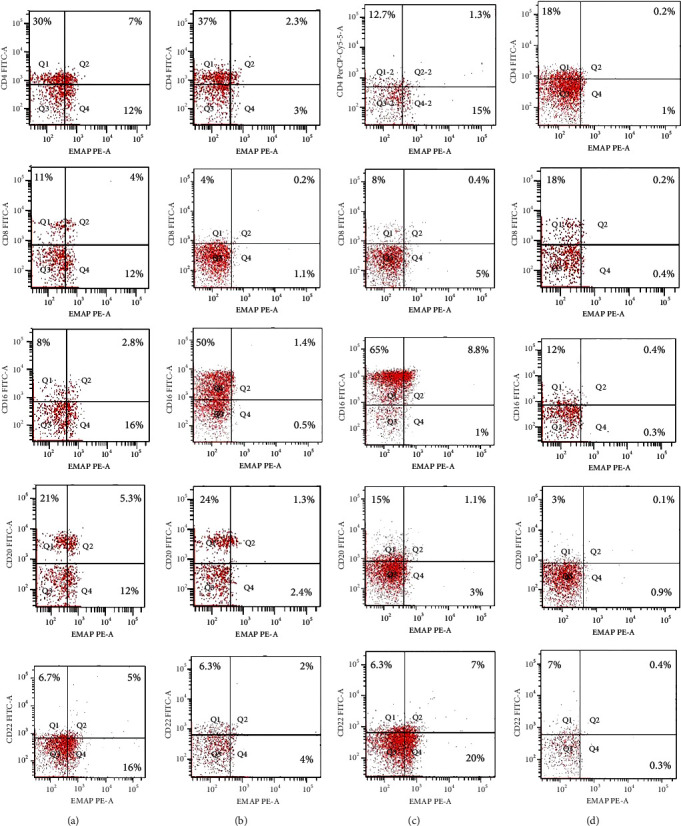
EMAP II is expressed on peripheral blood cells in NHL patients and normal controls. The cells were stained with FITC-labeled, PE-labeled and PerCP-Cy5.5-labeled antibodies. (a) Newly diagnosed NHL patients. (b) Patients with CR. (c) Patients with recurrence. (d) Flow cytometric dot plots of normal control.

EMAP II expression was examined in blood cells obtained from patients before and after therapy. In NHL, EMAP II was expressed dimly by peripheral blood cells. Newly diagnosed NHL subjects had a higher EMAP II+CD4+% than patients with CR [median: 7.4% (range 6.8-8.1) vs. 2.3% (range 1.5-2.7); *P* = 0.001] as well as a higher percentage of EMAP II+CD8+ [median: 4% (range 4-5) vs. 0.6 (range 0.4-0.7); *P* = 0.001]. The median percentage of EMAP II+CD16+ cells in newly diagnosed patients was 2.8% (range: 2.5-3.0) vs. 1.1% (range: 1.0-1.4) in patients with complete remission, *P* = 0.023. Measuring EMAP II expression in peripheral blood cells showed that EMAP II+CD20+% was higher in newly diagnosed patients than in patients with CR [median: 6% (range 5-6) vs. 1% (range 0.8-1.2%); *P* = 0.001]. Similarly, EMAP II+CD22+% was also increased in newly diagnosed patients compared to patients with CR [median 6.1: (5.9-6.6%) vs. 2.4: (2-3.5%); *P* = 0.001] (Figure 1 and Table 2). Gating of peripheral immune cells was shown in Supplementary Figure 1.

In relapse, patients had a higher percentage of EMAP II+CD16+ cells compared to pretherapy patients [median: 8% (range 6.2–8) vs. 2.8% (range: 2.5–3.0); *P* = 0.003]. Also, there was an increase in EMAP II+CD22+% within relapse group III compared to newly diagnosed group I but without significance (*P* > 0.05). Furthermore, when compared to pretherapy NHL subjects, EMAP II+CD4+%, EMAP II+CD8+%, and EMAP II+CD20+% were lower in NHL patients with recurrence (*P* = 0.001) (Figure 1 and Table 2).

EMAP II expression was examined in blood cells obtained from patients with relapse and CR. NHL subjects with relapse had a lower EMAP II+CD4+% than patients with CR [median: 0.7% (range 0.6–1.0) vs. 2.3% (range 1.5–2.7); *P* = 0.004] as well as a lower percentage of EMAP II+CD8+ in patients with disease recurrence than in CR [median: 0.2% (range 0.1–0.3) vs. 0.6 (range 0.4–0.7); *P* = 0.005]. The median percentage of EMAP II+CD16+ cells in relapsed patients was 8% (range: 6.2-8.8) compared to 1.1% in complete remission patients (range: 1.0-1.4), *P* < 0.001. Furthermore, the proportion of EMAP II+CD22+ was higher in patients with disease relapse than in patients with CR [median: 6.8% (range 6-7.3%) vs. 2.4% (range 2.0-3.5%); *P* < 0.001]. No differences were identified between NHL subjects with recurrence and patients with CR with regard to EMAP II+CD20+% (*P* > 0.05) (Table 2 and Figure 1).

**Figure 2:**
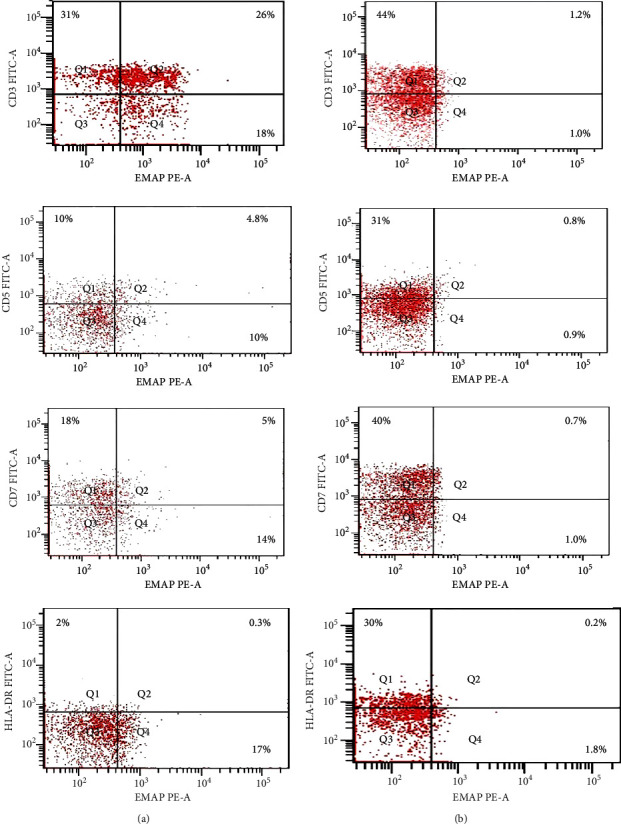
EMAP II expression in CD3, CD5, CD7, and HLA-DR cells: (a) newly diagnosed NHL patient; (b) representative flow cytometric dot plots of normal control.

EMAP II expression on CD3, CD5+, CD7+, and HLA-DR+ cells in 5 T-NHL patients was investigated. EMAP II was dimly positive in T-NHL, with a percentage of EMAP II+CD3+ positive cells above 20%. A high increase in EMAP+CD3+% was observed in newly diagnosed NHL patients compared to healthy volunteers [median: 24.3% (range: 24-26) and 1.3% (range: 1.3-1.4); *P* < 0.001, respectively]. The percentages of EMAP+CD5+ and EMAP II+CD7+ cells were higher in new T-NHL patients than in healthy controls (median = 2.9% vs. 0.2%; 3.5% vs. 0.3%; *P* < 0.001). However, no statistical significance was observed regarding EMAP II+HLADR+ percentages in this small group when compared to normal controls [median = 0.3% (range: 0.3-0.4) vs. 0.2% (range: 0.2-0.3); *P* > 0.05] (Figure 2, Supplementary Table 3).

### 3.3. EMAP II– and CD36–Coexpressing Cells in NHL

**Figure 3:**
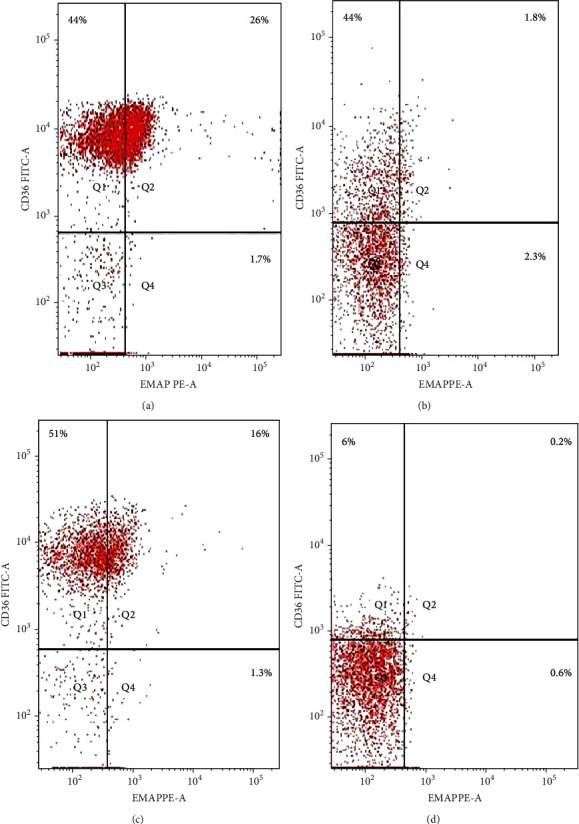
Flow cytometric detection of CD36 in EMAP+ lymphocytes. (a) NHL patient has not received treatment yet. (b) NHL patients achieved CR. (c) Patients with disease relapse. (d) Normal control.

The coexpression of EMAP II+ and CD36+ on peripheral lymphocytes was analyzed in patients with NHL and peripheral lymphocytes from healthy controls. In newly diagnosed patients, nearly all peripheral EMAP II+ lymphocytes coexpressed CD36, whereas in healthy controls, EMAP II+CD36+ expression was minimal [median 28.4% (range: 26.7-29.3) vs. 0.5% (range: 0.4-0.6); *P* < 0.001]. In complete remission, the median EMAP II+CD36+% was 2.2% (range: 2-2.47). Coexpression of CD36 and EMAP II was also higher in NHL subjects with relapse compared with patients with CR and healthy controls [median: 28.5% (range: 21.5-30.25); *P* < 0.001] (Figure 3).

### 3.4. EMAP II Expression Status (%) and LDH

All patients had their serum LDH levels checked, which is an important traditional prognostic sign for NHL. Regarding LDH levels, there was a high increase in LDH levels in NHL patients compared to healthy controls (*P* < 0.001). Contrary, there was a significant decrease in LDH among patients with CR compared to newly diagnosed NHL patients (*P* = 0.03). However, there were nonsignificant differences between NHL patients with relapse compared to newly diagnosed patients and patients with CR. The comparison of LDH levels between NHL subgroups is shown in Supplementary Table 4.

Table 3 shows that the percentage of EMAP II+CD16+, EMAP II+CD20+, and EMAP II+CD22+ cells was associated with LDH levels (*P* = 0.007, *P* = 0.025, and *P* = 0.042, respectively).

### 3.5. EMAP II Expression Status (%) and Clinical Criteria

Hepatomegaly and splenomegaly were associated with an increase in EMAP II+CD16+% (*P* = 0.002 and *P* = 0.003, respectively). Subjects with advanced high-risk disease (stages III and IV) had a higher EMAP II+CD16+% of cells than subjects with intermediate-risk disease (stage I-II) [median: 3.4% (range: 2-8) vs. 1.6% (range: 1.1-3.2); *P* = 0.004] Table 4. The results revealed that EMAP II+CD22+% cells in NHL subjects with advanced stage (stages III and IV) were higher compared with patients with intermediate-risk disease but with no significance [median: 6.3% (range: 3.6-7) % vs. 5.5% (range: 2.2-7) %; *P* = 0.218] (Table 4).

**Figure 4:**
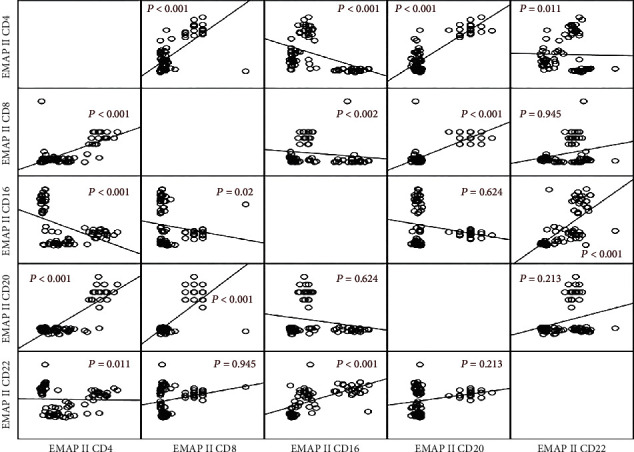
Correlations between EMAP II percentages on peripheral blood cells in NHL patients. *P* values are shown.

The correlation between EMAP II+ cells in NHL patients (Figure 4) was further investigated. EMAP II+CD4+% was positively associated with EMAP II+CD8+% and EMAP II+CD20+% of cells (*r* = 0.659, *P* < 0.001; *r* = 0.510, *P* < 0.001) but negatively associated with EMAP II levels on CD16+ and CD22+ cells (*r* = 0.445, *P* < 0.001; *r* = 0.284, *P* = 0.011). EMAP II+CD8+ percentages also had a negative relationship with EMAP II+CD16+ (*r* = 0.026, *P* = 0.02) and a positive relationship with EMAP II+CD20 (*r* = 0.453, *P* < 0.001). EMAP II+CD16+% had a significant positive association with EMAP II+CD22+ cell percentages (*P* < 0.001) (Figure 4).

### 3.6. Diagnostic Efficacy of EMAP II for NHL

**Figure 5:**
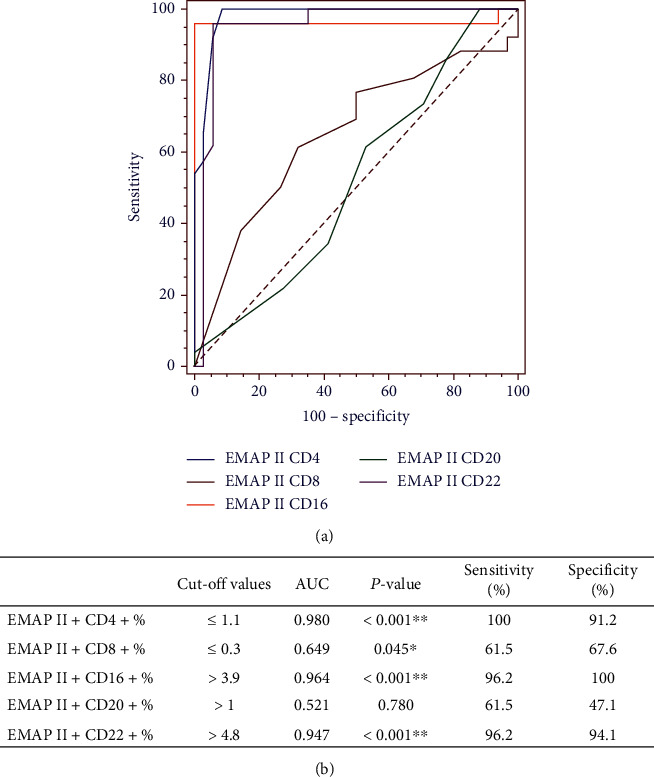
Diagnostic performances of percentages of EMAP II+CD4, EMAP II+CD8+, EMAP II+CD16+, EMAP II+CD20+, and EMAP II+CD22+ for identifying NHL patients with recurrence. (a) ROC curves were gained by curves at different cut-offs for NHL patients. (b) AUC (area under the curve) with cut-offs, sensitivity, and specificity for the markers. Significance (*P* < 0.05) is identified with ^∗^. High statistical significance (*P* < 0.001) is identified with ^∗∗^.

Receiver operating characteristic (ROC) curve analysis was performed for discriminating patients with recurrence, as shown in (Figure 5(a)). ROC curve analysis showed that the area under the ROC curve (AUC) of EMAP II+CD4+%, EMAP II+CD8+%, EMAP II+CD16+%, EMAP II+CD20+%, and EMAP II+CD22+% in peripheral blood was 0.980, 0.649, 0.964, 0.521, and 0.947 with cut-off value of ≤1.1, ≤0.3, >3.9, >1, and >4.8 being the most approximate index, respectively. In addition, based on the cut-off values, the specificity and sensitivity of EMAP II+CD4+% were 91.2% and 100%, respectively, while EMAP II+CD16+% were 100% and 96.2%, respectively. The specificity and sensitivity of EMAP II+CD22+% were 94.1% and 96.2%, respectively (Figure 5(b)).

**Figure 6:**
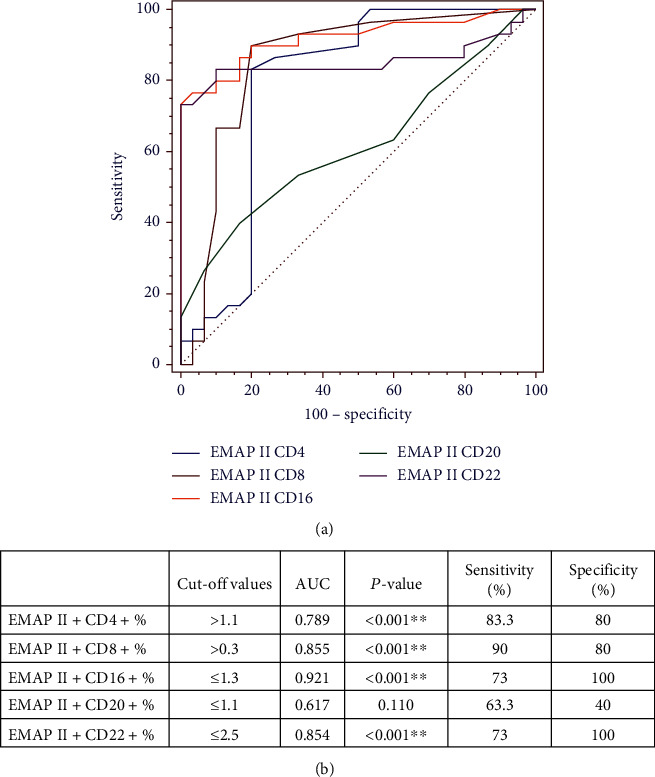
Diagnostic performances of percentages of EMAP II+CD4, EMAP II+CD8+, EMAP II+CD16+, EMAP II+CD20+, and EMAP II+CD22+ for identifying NHL patients with complete remission. (a) ROC curves were gained by curves at different cut-offs for NHL patients. (b) AUC (area under the curve) with cut-offs, sensitivity, and specificity for the markers. High statistical significance (*P* < 0.001) is identified with ^∗∗^.

Furthermore, ROC curves for differentiating patients with complete remission were determined. EMAP II+CD4+% AUC was 0.789 (*P* < 0.001). In addition, EMAP II+CD8+% showed an AUC of 0.855 (*P* < 0.001). EMAP II+CD16+% AUC was 0.921, while EMAP+CD22+% AUC was 0.854 (*P* < 0.001) (Figure 6(a)). These ROC curves indicated that EMAP II+CD16+% value of ≤1.3 and EMAP II+CD22+% value of ≤2.5 yielded a specificity and sensitivity of 100% and 73%, respectively, for discriminating patients with CR. For EMAP II+CD4+%, the cut-off with the maximum specificity and sensitivity for identifying CR was >1.1. Additionally, the specificity and sensitivity of EMAP II+CD8+% were 80% and 90%, respectively, while applying a cut-off level of >0.3 (Figure 6(b)).

## 4. Discussion

EMAP II expression has been demonstrated to affect tumor immune suppression and regulation [33, 46–48]. Serum EMAP II levels were elevated in NHL, suggesting EMAP II's role in NHL [49]. However, EMAP II expression on peripheral lymphocytes in tumors has not been evaluated, and its predictive and prognostic values are still not thoroughly investigated. The study assessed EMAP II expression on peripheral blood cells in NHL and further explored its association with disease outcomes and clinical data. The data revealed direct evidence that EMAP II was involved in the progression and development of NHL.

The results concur that newly diagnosed NHL patients had dim EMAP II expression in peripheral blood cells in NHL patients using flow cytometry. Flow cytometry analysis, especially when utilizing a strong fluorochrome like phycoerythrin, is a very sensitive approach for detecting dim antigen expression than immunohistochemistry. Using unstained cells and isotype controls clarified that the dim expression and low frequency of EMAP II might not be an artifact. Negligible EMAP II expression was detected in healthy controls, thus assuming a significant role for EMAP II in NHL pathogenesis. This study expanded the results of the previous study [49]. Overexpression of EMAP II/P43 was confirmed in patients with mantle cell lymphoma compared to normal cells [75]. Thus, EMAP II could potentially play a role in NHL pathogenesis.

EMAP II's role in cancer is not known. Some reports have shown that EMAP II is a tumor suppressor [27, 30, 76–78]. On the other hand, previous studies have indicated that EMAP II protein expression was identified in tumor cells [32, 33, 45, 47]. EMAP II transcripts have been found in a variety of human tissues as well as normal and malignant cell lines [11, 25]. However, there was no information regarding EMAP II expression in peripheral blood cells in tumors.

Subsequently, the results demonstrated EMAP II expression in peripheral blood cells in NHL subjects and normal volunteers. This is in line with the theory that all human normal and malignant cells express the intracellular 34 kDa form of EMAP II as a multisynthetase complex part in p43 form [12, 15]. EMAP II is released and processed under different circumstances, one of which is malignant transformation [11, 32, 33].

The mechanism by which EMAP II is subsequently present in peripheral blood cells is poorly understood. A previous report implied high serum EMAP II levels in subjects with NHL [49]. It is, therefore, possible that lymphoma cells express and release EMAP II and are subsequently found in the blood of NHL patients. EMAP II might act against normal lymphocytes. In Jurkat cells and peripheral blood mononuclear cells (PBMCs), EMAP II inhibited cell proliferation and DNA synthesis, inducing cell death. Native EMAP II expressed activated caspase 8 in Jurkats, causing cell apoptosis [45, 46]. It is presumed that lymphoma cells released EMAP II, facilitating EMAP II-induced apoptosis of lymphocytes. Future experiments are needed to confirm this theory.

Another scenario of increased EMAP II expression in blood cells is due to membrane translocation rather than increased EMAP II synthesis. EMAP II is translocated from inside the cell to the cell membrane through an unknown mechanism. The EMAP II 34 kDa form lacks a signal peptide required for translocation nor is it subjected to being glycosyl phosphatidyl inositol-anchored. Hypoxia increased EMAP II expression on the cell surface. The expression and release of plasminogen activator-1 and matrix metalloproteinases from tumor cells were upregulated by hypoxia [79, 80]. These enzymes might be engaged in EMAP II processing at the cell surface. Future experiments are needed to detect EMAP II expression in lymphoma cells and to explain these theories.

The previous study had shown that serum EMAP II could be positively associated with NHL progression [49]. This study demonstrated high EMAP II expression was observed in peripheral cells before treatment and decreased significantly after therapy. EMAP II may suppress antitumor immunity in newly diagnosed NHL, while treatment could induce a disruption in EMAP II signaling and expression. Another possibility is that the therapy induced the recruitment of EMAP II+ lymphocytes into the tumor area, leading to a decrease in the percentage of peripheral EMAP II+ cells. These results could be explained by the fact that EMAP II might have a part in the pathogenesis and progression of NHL. Future studies are required to describe the dysfunction of these cells.

EMAP II expression on CD16 and CD22 cells was significantly higher in a relapse in the current subject. NHL patients with relapse had lower EMAP II expression on CD4, CD8, and CD20 cells. Previous reports have demonstrated the correlation between high EMAP II expression and poor clinical outcomes or tumor metastasis [50]. This is concluded by using EMAP II to detect relapse, assuming longer endurance and earlier therapy.

This study analyzed EMAP II expression on peripheral CD3+, CD5+, CD7+, and HLA-DR+ in 5 T-NHL patients. EMAP II was identified on CD3+ cells in NHL patients. The high EMAP II expression on CD3+ cells in newly diagnosed NHL patients has clinically significant implications as it might provide a potential biomarker for NHL patients. Moreover, EMAP II was also present on CD5+ and CD7+ cells. EMAP was frequently expressed on CD3+ cells but was weak on CD5+ and CD7+ cells. Aberrant lack or dim expression of one or more pan-T antigens is required for T cell tumor diagnosis [81–86]. Previous studies reported the lack of CD5 and CD7 expression in T-NHL [85, 87]. In addition, the patients had decreased expression of HLA-DR on EMAP II+ cells compared to results in normal controls. Previously, it was shown that reduced HLA-DR expression is a powerful tool for tumor immune evasion [88]. It is possible that EMAP II could participate in suppressing host immunity in cancer. However, the group is too small to represent valuable statistical data.

CD36 is upregulated in patients with NHL [59, 60]. Studies of the EMAP II gene illustrated that EMAP II was associated with immune suppression in tumors [45, 47, 48]. However, no direct results were found to show whether EMAP II was upregulated on immune cells in NHL or whether EMAP II expression was related to CD36 expression. The results suggested that both CD36 and EMAP II were upregulated on peripheral blood cells in NHL patients and that more than 90% of EMAP II+ peripheral lymphocytes were CD36+, while there was no expression compared to peripheral lymphocytes from healthy controls. CD36 was found on less than 2% of normal CD8+ and CD4+ cells, 3% of normal CD19+ cells, and 4% of normal NK cells [61]. Circulating lymphocytes might express low levels of CD36 mRNA [89].

CD36 has a crucial role in immune suppression in cancer by enhancing T cell dysfunction and cancer progression [90, 91]. CD36 is found in various cell types and is thought to play a role in lipid transport in malignancies [92]. The differentiation and functioning of different types of T cells and the maintenance of immunological tolerance are all influenced by lipid metabolism [93]. Interestingly, a preceding study revealed that CD36-mediated cell death impairs CD8+ T antitumor activity and inhibits its functions [94]. Moreover, cell apoptosis was observed in CD8+ T cells with high CD36 expression. High CD36 expression in Treg cells initiates their survival and disables CD8+ T cell antitumor response [95]. EMAP II and CD36, which function as inhibitory molecules in T cell proliferation, could be linked to NHL pathogenesis. The roles of EMAP II and CD36 in the pathogenesis of NHL need further evaluation.

The study investigated an association between the percentages of EMAP II+ cells in NHL patients. Data revealed a significantly positive association of EMAP II expression on different cells, suggesting that EMAP II regulation was not cell-specific in NHL. As EMAP II is expressed on other cells like macrophages, monocytes, and dendritic cells, the level of EMAP II on these cells will be evaluated in future studies.

Further, the study revealed significant differences between LDH levels when NHL patients were compared to normal controls. The study demonstrated that percentages of EMAP II+CD16+, EMAPII+CD20+, and EMAP II+CD22+ cells had a significant positive association with elevated LDH levels, which implies the value of EMAP II expression on peripheral blood cells in NHL as a potential biomarker. The reported associations in these data support the correlation between higher levels of EMAP II expression and bad prognosis as elevated LDH levels, which are considered risk factors for poor performance and inferior overall survival [96].

The study also examined EMAP II expression on peripheral blood cells and the prognosis of NHL by evaluating EMAP II expression clinical data and identified that percentages of EMAP II+CD16+ were elevated in NHL patients with hepatomegaly or splenomegaly. Increased percentages of EMAP+CD16+ cells are linked to NHL stages 3 and 4, implying that EMAP II may play an important role in advanced tumor stages. High EMAP II levels were correlated with a worse prognosis in tumors [33, 49–51]. These findings suggest a potential poor prognostic influence of EMAP II in NHL and that high EMAP II expression might be associated with more aggressive or advanced disease. However, it is unknown how EMAP II might influence NHL progression and development. EMAP II might inhibit the activation and proliferation of lymphocytes. Future studies are required.

To the best of our knowledge, no previous reports analyzed ROC curves to assess EMAP II diagnostic performance in different peripheral blood cells in NHL. Previous reports investigated the diagnostic performance of different markers other than EMAP II in NHL [97]. According to the data, EMAP II+ cell percentages could be a valuable diagnostic for diagnosing NHL-related relapse and identifying individuals in complete remission. Hence, future studies with many patients might be helpful for the investigation of EMAP II expression.

The data revealed that the AUC of percentages of EMAP II+CD4+ cells for distinguishing NHL patients with recurrence was 0.98, and a cut-off for EMAP II + CD4 + % ≤ 1.1. The specificity and sensitivity of EMAP II+CD4+% for differentiating relapse in NHL were 91.2% and 100%, respectively. The AUC value of EMAP II+CD16+% for detecting patients with relapse was 0.964, which was higher than EMAP II+CD22+% (AUC = 0.947) and with a cut-off for EMAP II + CD16 + % > 3.9 and for EMAP II + CD122 + % > 4.8. The specificity and sensitivity of EMAP II + CD16 + % for differentiating relapse were 100% and 96.2%, respectively, while 94.1% and 96.2%, respectively, for EMAP II+CD22+%. These findings assume that EMAP II+CD16+% diagnostic performance might be superior to EMAP II+CD22+% in identifying NHL patients with recurrence.

In this study, the AUC of EMAP II+CD16+% for distinguishing NHL patients with complete remission from normal individuals was 0.921. It was higher than the EMAP II+CD22+% (AUC = 0.854) percentage, with a cut-off for EMAP II+CD16+% = 1.3%. The specificity and sensitivity of EMAP II+CD16+% and EMAP II+CD22+% for differentiating this subgroup were 100% and 73%, respectively. The AUC of EMAP II+CD4+% for identifying NHL with complete remission was higher than EMAP II+CD8+ (AUC = 0.855 and 0.789, respectively). Percentages of EMAP II+CD16+ had the best diagnostic performance for diagnosing complete remission in NHL.

Several limitations should be noted. The overall sample size of NHL cases, especially T-NHL cases, is relatively small; thus, further larger prospective longitudinal studies are required. Another limitation is the lack of EMAP II assessment on lymphoma cells. Future experiments are required to assess tumoral EMAP II expression in patients with lymphoma. Further studies are required to investigate the mechanistic insights of EMAP II expression in peripheral blood cells. Two-color staining might be considered a limitation of the study. Multicolor protocols will be followed in future experiments.

## 5. Conclusion

In conclusion, this study provides the first evidence of EMAP II expression on peripheral blood cells and highlights its prognostic value in NHL. Peripheral CD36 and EMAP II coexpression in NHL patients suggests that EMAP II expression might regulate tumor dissemination and identify NHL patients with more aggressive diseases. The data highlight and inform about the pathogenesis of NHL.

## Figures and Tables

**Table 1:** Clinical characteristics of non-Hodgkin's lymphoma (NHL) patients.

	*N* (%)
Group	
Group I (newly diagnosed)	20 (25.0%)
Group II (complete remission)	30 (37.5%)
Group III (relapse)	30 (37.5%)

Subtype	
DLBCL	59 (73.8%)
CLL	10 (12.5%)
FCL	7 (8.8%)
SCL	1 (1.2%)
MALT	2 (2.5%)
Missing	1 (1.2%)

B/T	
B	74 (92.5%)
T	3 (3.8%)
Mixed	2 (2.5%)
Plasma cell differentiation	1 (1.2%)

Hepatomegaly	
Positive	39 (48.8%)
Negative	41 (51.2%)

Splenomegaly	
Positive	49 (61.2%)
Negative	31 (38.8%)

Stage	
I	9 (11.3%)
II	26 (32.5%)
III	21 (26.2%)
IV	24 (30.0%)

*N*: number; DLBCL: diffuse large B-cell lymphoma; SCL: small lymphocytic lymphoma; CLL: chronic lymphocytic leukemia; FCL: follicular cell lymphoma; MALT: mucosa-associated lymphoid tissue lymphoma.

**Table 2:** Percentage of EMAP II in peripheral blood cells in non-Hodgkin's lymphoma (NHL) patients.

	Group I (newly diagnosed)	Group II (complete remission)	Group III (relapse)	*P* value		
	(*n* = 20)	(*n* = 30)	(*n* = 30)			
EMAP II+CD4+% *Median (IQR)*	7.4 (6.8-8.1)	2.3 (1.5-2.7)	0.7 (0.6-1)	<0.001^∗∗^		
				I vs. II	I vs. III	II vs. III
				0.001^∗^	0.001^∗^	0.004^∗^

EMAP II+CD8+% *Median (IQR)*	4 (4-5)	0.6 (0.4-0.7)	0.2 (0.1-0.3)	<0.001^∗∗^		
				I vs. II	I vs. III	II vs. III
				0.001^∗^	0.001^∗^	0.005^∗^

EMAP II+CD16+% *Median (IQR)*	2.8 (2.5-3)	1.1 (1-1.4)	8 (6.2-8.8)	<0.001^∗∗^		
				I vs. II	I vs. III	II vs. III
				0.023^∗^	0.003^∗^	<0.001^∗∗^

EMAP II+CD20+% *Median (IQR)*	6 (5-6)	1 (0.8-1.2)	1.1 (1-1.3)	<0.001^∗∗^		
				I vs. II	I vs. III	II vs. III
				0.001^∗^	0.001^∗^	0.786

EMAP II+CD22+% *Median (IQR)*	6.1 (5.9-6.6)	2.4 (2-3.5)	6.8 (6-7.3)	<0.001^∗∗^		
				I vs. II	I vs. III	II vs. III
				0.001^∗^	>0.99	<0.001^∗∗^

*N*: number; IQR: interquartile range. Significance (*P* < 0.05) is identified with ^∗^. High statistical significance (*P* < 0.001) is identified with ^∗∗^. Total events are 10,000 events. The percentages of positive cells were assessed from the lymphocyte gate. Kruskal-Wallis test was used, followed by Dunn's test with Bonferroni correction.

**Table 3:** Correlation of EMAP II expression in different blood cells and LDH levels in NHL patients.

	LDH (U/L)	
	Rho	*P*
EMAP II+CD4+%	-0.03	0.793
EMAP II+CD8+%	-0.026	0.818
EMAP II+CD16+%	0.299	0.007^∗^
EMAP II+CD20+%	0.250	0.025^∗^
EMAP II+CD22+%	0.227	0.042^∗^

NHL: non-Hodgkin's lymphoma; LDH: lactate dehydrogenase. Statistically, significance (*P* < 0.05) is identified with ^∗^.

**Table 4:** Correlation between percentages of EMAP II+CD4+, EMAP II+CD8+, EMAP II+CD16+, EMAP II+CD20+, and EMAP II+CD22+ and clinical characteristics of NHL patients.

	*N*	EMAP II+CD4+%	EMAP II+CD8+%	EMAP II+CD16+%	EMAP II+CD20+%	EMAP II+CD22+%
		Median (*IQR*)	Median (*IQR*)	Median (*IQR*)	Median (*IQR*)	Median (*IQR*)
Hepatomegaly						
Positive	39	1.6 (0.7-7.3)	0.5 (0.1-4)	3.4 (2.4-8)	1.3 (1-5)	6.3 (4.5-7)
Negative	41	2.4 (1.1-3.9)	0.6 (0.4-1)	1.5 (1.1-3.2)	1.1 (0.9-1.3)	3.7 (2.2-7)
*P* value		0.689	0.615	0.002^∗^	0.062	0.067
Splenomegaly						
Positive	49	2 (0.8-6.4)	0.5 (0.2-3)	3.1 (2-8)	1.2 (1-1.5)	6.1 (3.5-7)
Negative	31	2.5 (1.4-6.1)	0.6 (0.4-3)	1.6 (1.1-3.2)	1.2 (0.9-5)	5 (2.4-6.7)
*P* value		0.335	0.264	0.003^∗^	0.662	0.201
Stage						
Stage I/II	35	2.5 (1.4-6.1)	0.7 (0.4-3)	1.6 (1.1-3.2)	1.2 (0.9-5)	5.5 (2.2-7)
Stage III/IV	45	1.8 (0.7-6.4)	0.4 (0.1-3)	3.4 (2-8)	1.2 (1-1.5)	6.3 (3.6-7)
*P* value		0.098	0.076	0.004^∗^	0.438	0.218

NHL: non-Hodgkin's lymphoma; IQR: interquartile range. Significant statistical differences (*P* < 0.05) are identified with asterisks (^∗^).

## Data Availability

The datasets for this study are available from the corresponding authors upon reasonable request.

## Abbreviations

AIMP1: Aminoacyl-tRNA synthetase-interacting multifunctional protein 1
ARS: Aminoacyl-tRNA synthetase
AUC: Area under the ROC curve
CD36: Cluster of differentiation 36
CHOP: Cyclophosphamide, hydroxydaunorubicin, oncovin, prednisone
CR: Complete remission
EMAP II: Endothelial monocyte activating polypeptide-II
LDH: Lactate dehydrogenase
NHL: Non-Hodgkin lymphomas
PBMCs: Peripheral blood mononuclear cells
PBS: Phosphate-buffered solution
ROC curve: Receiver operating characteristic curve
TNF-alpha: TNF-alpha.

## Supplementary Materials

Supplementary Materials Supplementary Table 1: **c**omparison between NHL patients and healthy control as regards demographic data. Supplementary Table 2: Percentage of EMAP II in peripheral blood cells in Non-Hodgkin's Lymphoma (NHL) patients and healthy controls. Supplementary Table 3: percentage of EMAP II in peripheral immune cells in 5 T-NHL patients. Supplementary Table 4: comparison of LDH levels between NHL patients. Supplementary Figure 1: gating is used to detect cells for analysis.
