# Supplementary material for: EMAP II Expression Is Increased on Peripheral Blood Cells from Non-Hodgkin Lymphoma
Source: J Immunol Res. 2022 Sep 12;2022:7219207. doi: 10.1155/2022/7219207 (PMC9484964; doi:10.1155/2022/7219207)
Supplement: Supplementary Materials — Supplementary Table 1: comparison between NHL patients and healthy control as regards demographic data. Supplementary Table 2: Percentage of EMAP II in peripheral blood cells in Non-Hodgkin's Lymphoma (NHL) patients and healthy controls. Supplementary Table 3: percentage of EMAP II in peripheral immune cells in 5 T-NHL patients. Supplementary Table 4: comparison of LDH levels between NHL patients. Supplementary Figure 1: gating is used to detect cells for analysis. [file 7219207.f1.docx]

**Supplementary Table 1:** Comparison between NHL patients and healthy control as regards demographic data.

|  | **NHL**  **(n=80)** | **Control**  **(n=30)** | ***P*-value** |
| --- | --- | --- | --- |
| **Sex** |  |  |  |
| Male | 42 (52.5%) | 18 (60.0%) | 0.482 |
| Female | 38 (47.5%) | 12 (40.0%) |  |
| **Age**  Mean± SD  (Range) | 45.3±15.7  (8-81) | 44.5±15  (21-70) | 0.816 |

NHL: Non-Hodgkin lymphoma; N: Number.

**Supplementary Table 2:** Comparison between NHL patients and healthy controls as regards demographic data.

|  | **NHL** | **Control** | ***P*-value** |
| --- | --- | --- | --- |
|  | **(n=80)** | **(n=30)** |  |
| **EMAP II+CD4+%**  *Median (IQR)* | 2.3 (1-6.3) | 1 (0.6-1) | **< 0.001**** |
| **EMAP II+CD8+%**  *Median (IQR)* | 0.6 (0.2-3) | 0.5 (0.4-0.8) | 0.911 |
| **EMAP II+CD16+%**  *Median (IQR)* | 3 (1.2-7) | 0.5 (0.3-0.6) | **< 0.001**** |
| **EMAP II+CD20+%**  *Median (IQR)* | 1.2 (1-2.8) | 0.5 (0.4-0.8) | **< 0.001**** |
| **EMAP II+CD22+%**  *Median (IQR)* | 6 (2.5-7) | 2 (2-2) | **< 0.001**** |

NHL: Non-Hodgkin lymphoma; N: Number; IQR: Inter quartile range. Total events are 10 000 events. The percentages of positive cells were assessed from the lymphocyte gate. High statistically significance (*P* < 0.001) is identified with **.

**Supplementary Table 3:** Percentage of EMAP II in peripheral immune cells in 5 T-NHL patients.

|  | **Patient I** | **Patient II** | **Patient III** | **Patient IV** | **Patient V** |
| --- | --- | --- | --- | --- | --- |
|  |  |  |  |  |  |
| **EMAP II+CD3+%** | **26%** | **29%** | **24%** | **24.3%** | **20.8%** |
| **EMAP II+CD5+%** | **2.8%** | **2.9%** | **2.7%** | **3%** | **4.8%** |
| **EMAP II+CD7+%** | **4.5%** | **3.3%** | **3.2%** | **5%** | **3.5%** |
| **EMAP II+HLADR+%** | **0.4%** | **0.3%** | **0.4%** | **0.4%** | **0.2%** |

NHL: Non-Hodgkin lymphoma. Total events are 10 000 events. The percentages of positive cells were assessed from the lymphocyte gate.

**Supplementary Table 4:** Comparison of LDH levels between NHL patients.

|  | **Group I**  **(Newly diagnosed)** | **Group II (complete remission)** | **Group III (relapse)** | **Controls** | **Kruskal Wallis**  ***P*-Value** | | |
| --- | --- | --- | --- | --- | --- | --- | --- |
|  | **(n=20)** | **(n=30)** | **(n=30)** |  |  |  |  |
| **LDH (U/L)**  *Median (IQR)* | 700.5 (485.5-1034) | 352 (280-620) | 716 (520-820) | 288 (250 --315) | **< 0.001****  ***I vs. II I vs. III II vs. III*** | | |
|  |  |  |  |  | **0.03*** | **> 0.05** | **> 0.05** |

NHL: Non-Hodgkin lymphoma; N: Number; IQR: Interquartile range; LDH: Lactate dehydrogenase. Statistically significance (*P* < 0.05) is identified with *. High statistically significance (*P* < 0.001) is identified with **.


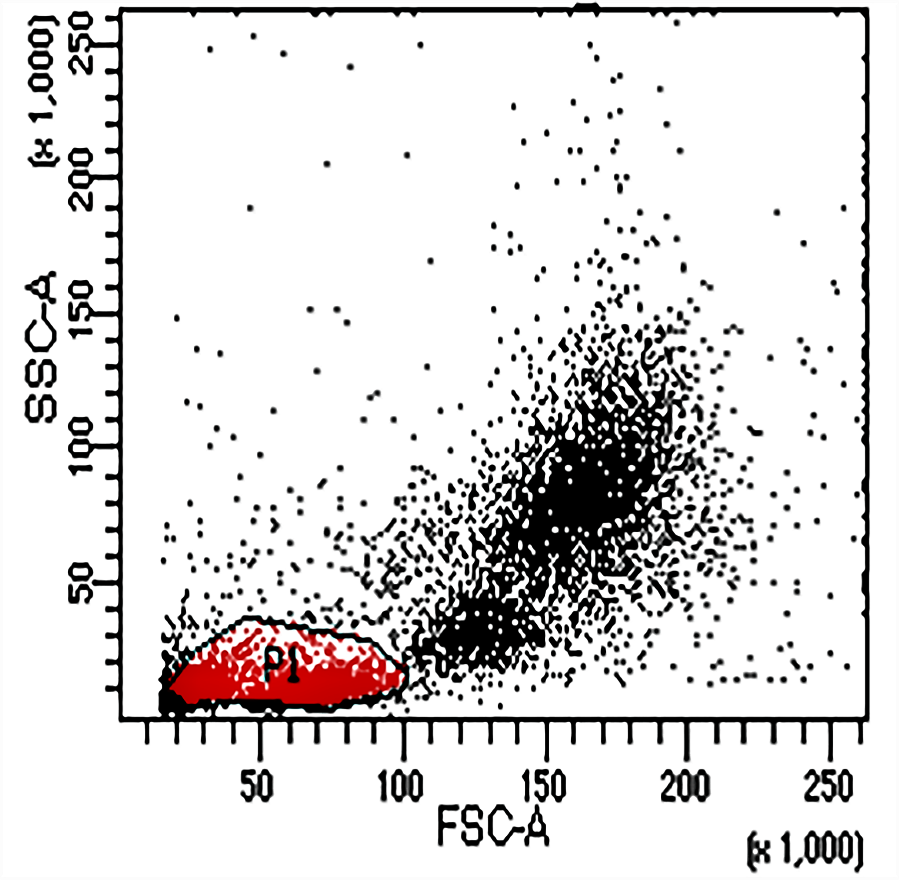


**Supplementary Figure 1:** Gating is used to detect cells for analysis.
